# Machine learning consensus clustering for inflammatory subtype analysis in stroke and its impact on mortality risk: a study based on NHANES (1999–2018)

**DOI:** 10.3389/fneur.2025.1562247

**Published:** 2025-04-10

**Authors:** Zhang Chunjuan, Wang Yulong, Zhou Xicheng, Ma Xiaodong

**Affiliations:** Haiyan People’s Hospital, Jiaxing, Zhejiang, China

**Keywords:** stroke, machine learning, consensus cluster, inflammation subtype, neutrophil-percentage-to-albumin ratio, systemic inflammatory response index

## Abstract

**Background:**

Our study aims to utilize unsupervised machine learning methods to perform inflammation clustering on stroke patients via novel CBC-derived inflammatory indicators (NLR, PLR, NPAR, SII, SIRI, and AISI), evaluate the mortality risk among these different clusters and construct prognostic models to provide reference for clinical management.

**Methods:**

A cross-sectional analysis was conducted using data from stroke participants in the U.S. NHANES 1999–2018. Weighted multivariate logistic regression was used to construct different models; consensus clustering methods were employed to subtype stroke patients based on inflammatory marker levels; LASSO regression analysis was used to construct an inflammatory risk score model to analyze the survival risks of different inflammatory subtypes; WQS regression, Cox regression, as well as XGBoost, random forest, and SVMRFE machine learning methods were used to screen hub markers which affected stroke prognosis; finally, a prognostic nomogram model based on hub inflammatory markers was constructed and evaluated using calibration and DCA curves.

**Results:**

A total of 918 stroke patients with a median follow-up of 79 months and 369 deaths. Weighted multivariate logistic regression analysis revealed that high SIRI and NPAR levels were significantly positively correlated with increased all-cause mortality risk in stroke patients (*p* < 0.001), independent of potential confounders; Consensus clustering divided patients into two inflammatory subgroups via SIRI and NPAR, with subgroup 2 having significantly higher markers and mortality risks than subgroup 1 (*p* < 0.001); LASSO regression analysis showed subgroup 2 had higher risk scores and shorter overall survival than subgroup 1 [HR, 1.99 (1.61–2.45), *p* < 0.001]; WQS regression, Cox regression, and machine learning methods identified NPAR and SIRI as hub prognostic inflammatory markers; The nomogram prognostic model with NPAR and SIRI demonstrated the best net benefit for predicting 1, 3, 5 and 10-year overall survival in stroke patients.

**Conclusion:**

This study shows NPAR and SIRI were key prognostic inflammatory markers and positively correlated with mortality risk (*p* < 0.001) for stroke patients. Patients would been divided into 2 inflammatory subtypes via them, with subtype 2 having higher values and mortality risks (*p* < 0.001). It suggests that enhanced monitoring and management for patients with high SIRI and NPAR levels to improve survival outcomes.

## 1 Introduction

Stroke, also termed cerebrovascular accident (CVA), is characterized by rapidly developing neurological deficits due to sudden cerebral blood flow disruption, leading to long-term disability or mortality. As a leading global cause of disability and death (1), stroke affects approximately 15 million individuals annually, with a substantial proportion experiencing recurrent events or persistent functional impairments. The inflammatory cascade plays a dual role in post-stroke pathophysiology, mediating both secondary neuronal injury and repair processes (2). While inflammation has emerged as a promising therapeutic target, the clinical benefits of systemic anti-inflammatory interventions remain controversial. Consequently, stratifying acute ischemic stroke (AIS) patients based on inflammatory heterogeneity may enhance pathophysiological understanding and enable tailored therapeutic modulation of neuroinflammation, thereby optimizing cerebral protection and functional recovery.

Complete blood count (CBC)-derived inflammatory indices—including neutrophil-to-lymphocyte ratio (NLR), platelet-to-lymphocyte ratio (PLR), neutrophil percentage-to-albumin ratio (NPAR), systemic inflammation response index (SIRI), systemic immune-inflammation index (SII), and aggregate index of systemic inflammation (AISI)—provide integrative prognostic insights by quantifying interactions among platelets, neutrophils, and lymphocytes. These indices have demonstrated diagnostic and prognostic utility across multiple diseases. In AIS, compelling evidence highlights their clinical relevance: NLR independently predicts 90-day functional outcomes (3), while NLR, PLR, lymphocyte-to-monocyte ratio (LMR), and SIRI correlate with in-hospital mortality, length of stay (4), and 3-month disability rates (5). Notably, these CBC-based biomarkers reflect real-time inflammatory dynamics through routine hematological testing, offering practical advantages over conventional cytokine assays. Their integration into AIS subtyping frameworks may thus facilitate precision medicine by identifying high-risk phenotypes amenable to targeted immunomodulation.

Unsupervised machine learning (ML), a statistical approach that identifies latent patterns by analyzing the underlying structures of unlabeled data, has demonstrated unique value in medical research for classification and risk stratification (6). As a pivotal branch of unsupervised ML, consensus clustering enables precise phenotypic classification by iteratively validating multidimensional feature heterogeneity across patient populations, independent of outcome variables. This methodology has been successfully applied to elucidate disease mechanisms and optimize therapeutic strategies across various conditions (7–10). Notably, its potential is emerging in stroke research. For instance, Yang et al. (11) classified AIS patients into three molecular subtypes based on peripheral blood monocyte transcriptomic profiles, revealing that the high-inflammatory-response subtype exhibited a significantly elevated risk of hemorrhagic transformation, providing critical insights for timing immunomodulatory therapies. Similarly, Cui et al. (12) integrated clinical and neuroimaging data using unsupervised ML to identify a subgroup showing superior therapeutic responses to a “statin combined with repetitive transcranial magnetic stimulation” regimen. However, existing studies have yet to systematically explore the role of CBC (complete blood count)-derived inflammatory indices (e.g., SIRI, NPAR) in AIS subtyping. These indices not only dynamically reflect key inflammatory pathways, such as neutrophil–platelet interactions, but also offer practical advantages for point-of-care testing.

This study applies consensus clustering to resolve inflammatory heterogeneity in AIS, aiming to achieve dual objectives: (1) identifying pathophysiologically meaningful stroke inflammatory subtypes based on CBC-derived inflammatory indices, and (2) constructing interpretable ML prognostic models to quantify mortality risk disparities across subtypes. Ultimately, this work seeks to provide evidence-based guidance for anti-inflammatory therapy optimization and monitoring frequency stratification (e.g., dynamic inflammation monitoring in high-risk subtypes), thereby facilitating the clinical translation of precision medicine in AIS management.

## 2 Methods

### 2.1 Data source

The National Health and Nutrition Examination Survey (NHANES) database is dedicated to the systematic collection of data pertaining to the health and nutritional status of households in the United States. It encompasses a wide array of information, including demographic characteristics, dietary assessments, results from physical examinations, responses to questionnaires, laboratory findings, and data with restricted access. The database utilizes a sophisticated stratified, multistage clustered sampling technique to ensure that the statistical sample accurately reflects the broader U.S. population. This research was sanctioned by the Ethics Review Committee of the National Center for Health Statistics, and informed consent was secured from all participants through signed consent forms. Comprehensive details regarding the publicly available NHANES research design and data can be found at https://www.cdc.gov/nchs/nhanes/.

### 2.2 Study population

This cohort study examined data from the continuous National Health and Nutrition Examination Survey (NHANES), which was conducted between 1999 and 2018. Participants under the age of 20, those with incomplete complete blood count (CBC) parameters, individuals lacking specific information regarding stroke, and those with missing critical covariate and follow-up data were excluded from the analysis. Consequently, the final sample comprised 918 patients diagnosed with stroke. Given the inclusion of hematological parameters in our study, we employed mobile examination center (MEC) weights for the data analysis. The weight calculation for the cohorts from 1999 to 2000 and 2001–2002 was determined using the formula 2/10 × wtmec4yr, whereas for the cohorts from 2003 to 2018, the formula applied was 1/10 × wtmec2yr.

### 2.3 Definition of inflammatory indices

The inflammatory markers evaluated in this research included the neutrophil-to-lymphocyte ratio (NLR), platelet-to-lymphocyte ratio (PLR), neutrophil percentage-albumin ratio (NPAR), systemic immune-inflammation index (SII), systemic inflammatory response index (SIRI), and aggregate index of systemic inflammation (AISI), all of which are derived from standard complete blood count (CBC) tests. The formulas utilized for the computation of these ratios are as follows: NLR = Neutrophil count (NC) / Lymphocyte count (LC); PLR = Platelet count (PC) / Lymphocyte count (LC); SII = (Platelet count (PC) × Neutrophil count (NC)) / Lymphocyte count (LC); NPAR = (Neutrophil percentage of total white blood cell count (%) × 100) / Albumin (g/dL); SIRI = (Neutrophil count (NC) × Monocyte count (MC)) / Lymphocyte count (LC); AISI = (Neutrophil count (NC) × Platelet count (PC) × Monocyte count (MC)) / Lymphocyte count (LC).

### 2.4 Covariates

The covariates utilized in this research included age, race, and ethnicity, which were categorized into the following groups: Mexican American, other Hispanic, non-Hispanic White, non-Hispanic Black, and other races. Educational attainment was classified into three distinct categories: individuals with less than a high school education, high school graduates, and those with education beyond high school. Family income was stratified into two categories: low income (less than 1.3 times the federal poverty level) and high income (greater than 3.5 times the federal poverty level). Smoking status was delineated between current smokers—defined as individuals who have smoked 100 or more cigarettes in their lifetime—and non-smokers, which included those who have smoked 100 cigarettes or fewer or who have never smoked. Alcohol consumption was operationally defined as the consumption of at least 12 alcoholic beverages within any year of the participant’s life. The diagnosis of diabetes was established based on one or more of the following criteria: confirmation by a physician or healthcare professional; a fasting blood glucose level of 126 mg/dL or higher; an HbA1c percentage of 6.5% or greater; or the use of diabetes medications, including insulin.

### 2.5 Statistical analysis

All statistical analyses were conducted with consideration of the intricate design of the National Health and Nutrition Examination Survey (NHANES). In the table detailing baseline characteristics, continuous variables were presented as weighted means accompanied by 95% confidence intervals (CIs), while categorical variables were expressed as weighted percentages with corresponding 95% CIs. To evaluate differences between groups, weighted linear regression and weighted chi-square tests were employed.

To investigate the relationships between six inflammatory markers and the risk of all-cause mortality in stroke patients, three logistic regression models were utilized. NPAR and SIRI were selected for consensus clustering subtype analysis, and LASSO regression was applied to develop a risk score model aimed at analyzing the mortality risk associated with different subtypes. Consensus clustering was implemented using K-means clustering with Euclidean distance through the ConsensusClusterPlus package (1.64.0). Subsequently, weighted quantile sum (WQS) regression models were employed to estimate the combined effects of the six inflammatory markers, allowing for the identification of primary markers through the calculated WQS index. Additionally, methods such as XGBoost, random forest (RF) and support vector machine recursive feature elimination (SVMRFE) were utilized to select key prognostic inflammatory markers. Ultimately, a prognostic nomogram model was developed based on the identified key inflammatory prognostic markers. The predictive accuracy of this nomogram was evaluated using calibration curves, while decision curve analysis (DCA) was conducted to assess the potential benefits to patients derived from the model.

## 3 Results

### 3.1 Study cohort selection

**Figure 1:**
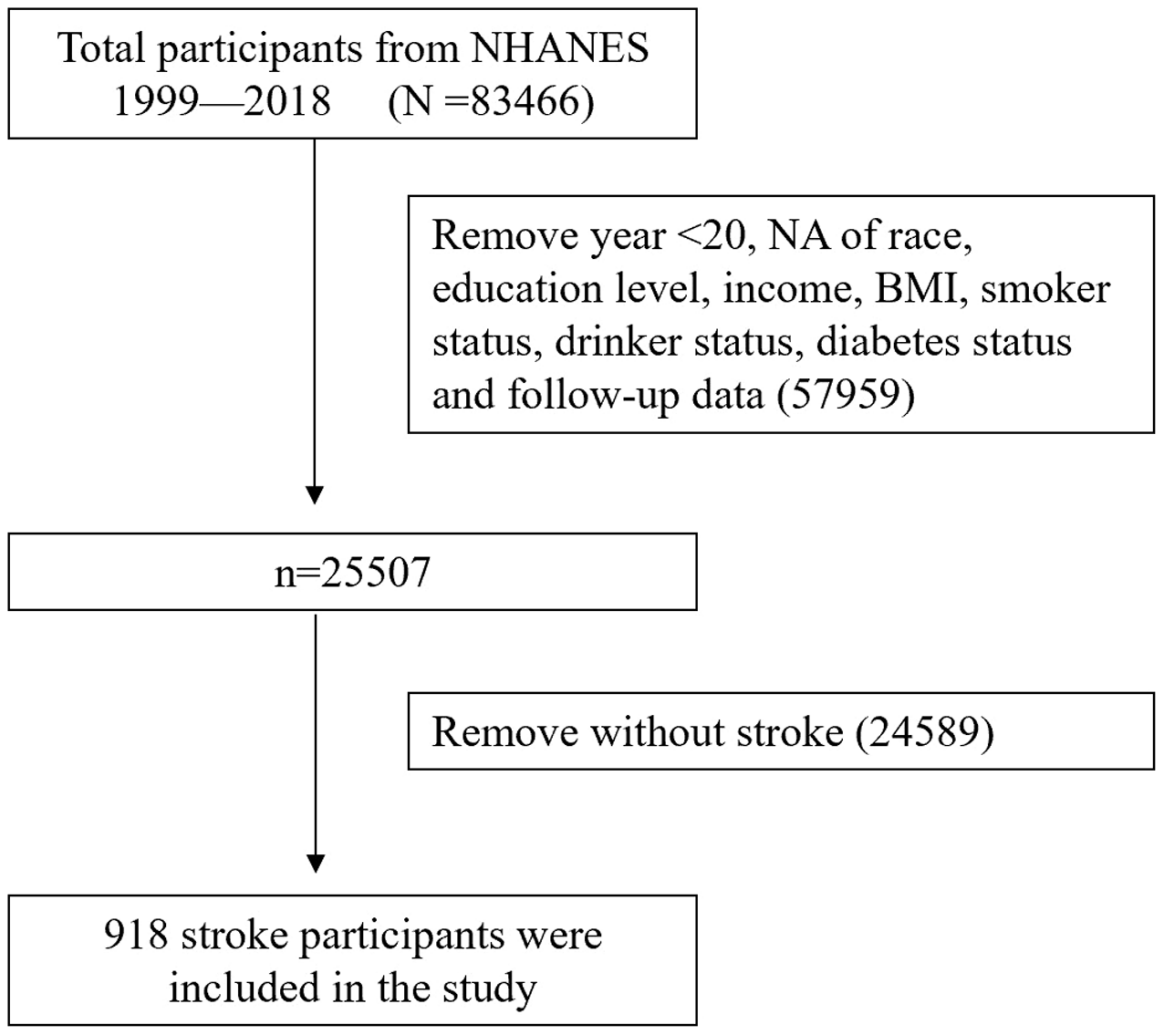
Study population screening flowchart.

Following the exclusion of participants lacking comprehensive primary variable data and mortality status information during the follow-up period from the NHANES database, a total of 918 individuals aged 20 years and older who had experienced a stroke were identified. The flowchart illustrating the study’s inclusion and exclusion criteria is depicted in Figure 1.

### 3.2 Baseline characteristics of the study cohort

Table 1 presents the baseline characteristics of the study cohort. Over a median follow-up period of 79 months, a total of 369 deaths were recorded, with deceased individuals being a significantly older (*p* < 0.001). in addition to household income, smoking status, and diabetes status, there were marked differences in the distributions of sociodemographic, behavioral, and health-related characteristics between the two groups. Furthermore, among the six inflammatory indicators assessed, the death group presented significantly greater NLR, NPAR, SIRI, and AISI values than the survival group (*p* < 0.001).

**Table 1:** Baseline characteristics of study participants in stroke patients, weighted.

	Stroke			
Characteristic	Overall, *N* = 918^1^	Alive, *N* = 549^1^	Dead, *N* = 369^1^	*p* value^2^
Age	67.0 (56.0, 77.0)	62.0 (51.0, 69.0)	77.0 (68.0, 80.0)	<0.001
Gender				0.4
Female	465 (56%)	305 (57%)	160 (54%)	
Male	453 (44%)	244 (43%)	209 (46%)	
Race				<0.001
Mexican American	85 (4.4%)	60 (5.1%)	25 (3.1%)	
Non-Hispanic Black	234 (13%)	160 (15%)	74 (9.8%)	
Non-Hispanic White	494 (73%)	248 (67%)	246 (82%)	
Other Hispanic	56 (2.8%)	43 (3.6%)	13 (1.4%)	
Other Race	49 (6.9%)	38 (8.8%)	11 (3.4%)	
Education				0.002
Above high school	355 (45%)	234 (50%)	121 (37%)	
Below high school	141 (9.9%)	77 (8.0%)	64 (14%)	
High school	422 (45%)	238 (42%)	184 (50%)	
Income				0.2
Poverty	227 (20%)	151 (22%)	76 (17%)	
Richer	691 (80%)	398 (78%)	293 (83%)	
BMI	29 (25, 34)	30 (25, 34)	28 (24, 32)	0.001
Drinker	608 (69%)	372 (73%)	236 (60%)	0.005
Smoker	259 (28%)	176 (31%)	83 (22%)	0.050
Hypertension	704 (75%)	404 (71%)	300 (81%)	0.003
Diabetes				0.8
Diabetes	343 (33%)	197 (32%)	146 (35%)	
Normal	275 (33%)	168 (34%)	107 (32%)	
Prediabetes	300 (34%)	184 (34%)	116 (33%)	
NLR	2.19 (1.69, 3.06)	2.08 (1.65, 2.74)	2.42 (1.81, 3.45)	0.004
PLR	123 (94, 155)	122 (95, 151)	125 (92, 168)	0.5
NPAR	145 (128, 160)	141 (125, 157)	151 (132, 171)	0.001
SII	525 (361, 730)	501 (353, 711)	558 (384, 781)	0.12
SIRI	1.26 (0.86, 1.95)	1.20 (0.82, 1.82)	1.53 (1.00, 2.17)	<0.001
AISI	299 (187, 450)	279 (183, 427)	348 (200, 514)	0.004

^1^Median (Q1, Q3); *n* (unweighted) (%). ^2^Design-based Kruskal-Wallis test; Pearson’s X^2^: Rao-Scott adjustment.

### 3.3 The associations between inflammatory markers and all-cause mortality in stroke patients

Table 2 presents the findings from the weighted multivariable logistic regression analysis. In Model 1, elevated levels of four inflammatory markers were found to be significantly correlated with an increased risk of all-cause mortality among stroke patients, particularly within the higher SIRI group, which exhibited an odds ratio (OR) of 1.49 (95% confidence interval [CI], 1.25–1.77). In Model 2, following adjustments for additional covariates including age, sex, race, education level, and alcohol consumption, the risk associated with three inflammatory markers remained significantly elevated, with SIRI demonstrating an OR of 1.29 (95% CI, 1.05–1.57). In Model 3, after controlling for all covariates, NPAR and SIRI, continued to exhibit a significant positive association with the risk of all-cause mortality in stroke patients (*p* < 0.05).

**Table 2:** Stepped logistic regression models showing the association between inflammation indicators and the odds of stroke patients.

Indicators	Model 1			Model 2			Model 3		
	OR^1^	95% CI^1^	*p*-value	OR^1^	95% CI^1^	*p*-value	OR^1^	95% CI^1^	*p*-value
NLR	1.24	1.06, 1.45	0.008	1.16	0.99, 1.35	0.066	1.14	0.99, 1.31	0.077
PLR	1.00	1.00, 1.00	0.200	1.00	1.00, 1.00	0.700	1.00	1.00, 1.00	0.600
NPAR	1.01	1.00, 1.02	0.002	1.01	1.00, 1.02	0.006	1.01	1.00, 1.02	0.010
SII	1.00	1.00, 1.00	0.120	1.00	1.00, 1.00	0.200	1.00	1.00, 1.00	0.300
SIRI	1.49	1.25, 1.77	<0.001	1.29	1.05, 1.57	0.015	1.24	1.02, 1.51	0.029
AISI	1.00	1.00, 1.00	0.003	1.00	1.00, 1.00	0.049	1.00	1.00, 1.00	0.090

### 3.4 KM survival curve analysis

**Figure 2:**
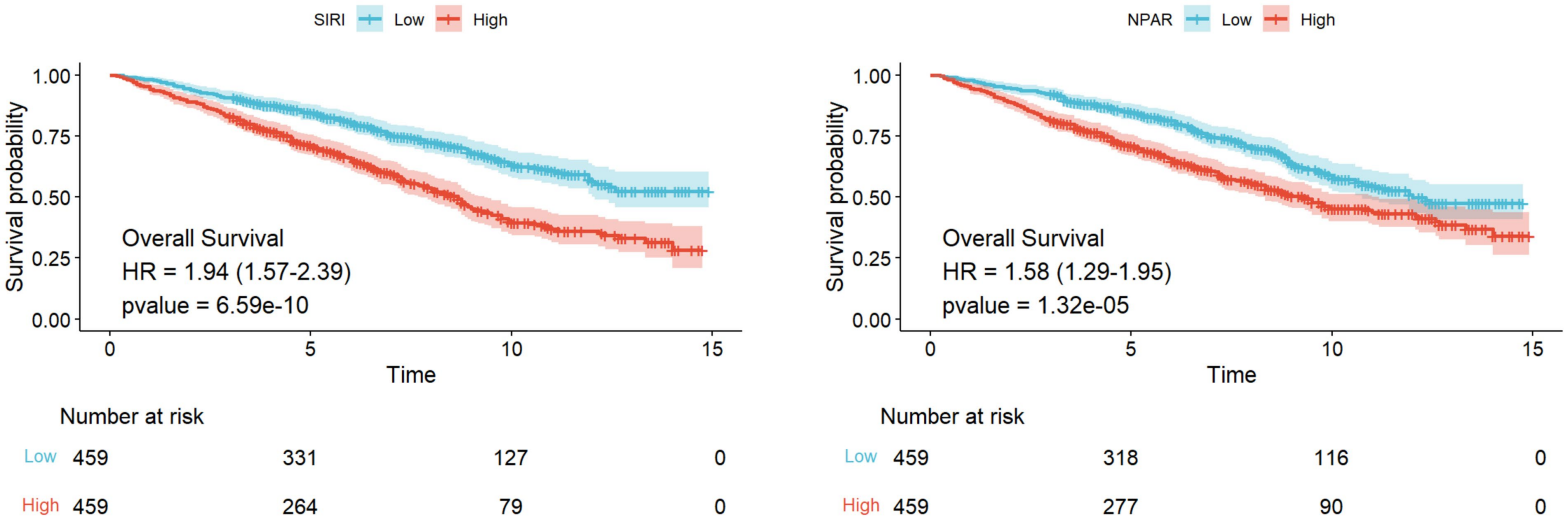
Kaplan–Meier plotter of SIRI and NPAR.

Figure 2 shows the results of the Kaplan–Meier plotter curve analysis, which demonstrated that higher expression levels of SIRI (hazard ratio [HR], 1.94 [1.57–2.39], *p* < 0.001) and NPAR (HR, 1.58 [1.29–1.95], *p* < 0.001) are associated with a significant reduction in the survival probability of patients who have experienced a stroke.

### 3.5 Construction of inflammation-related subtypes

**Figure 3:**
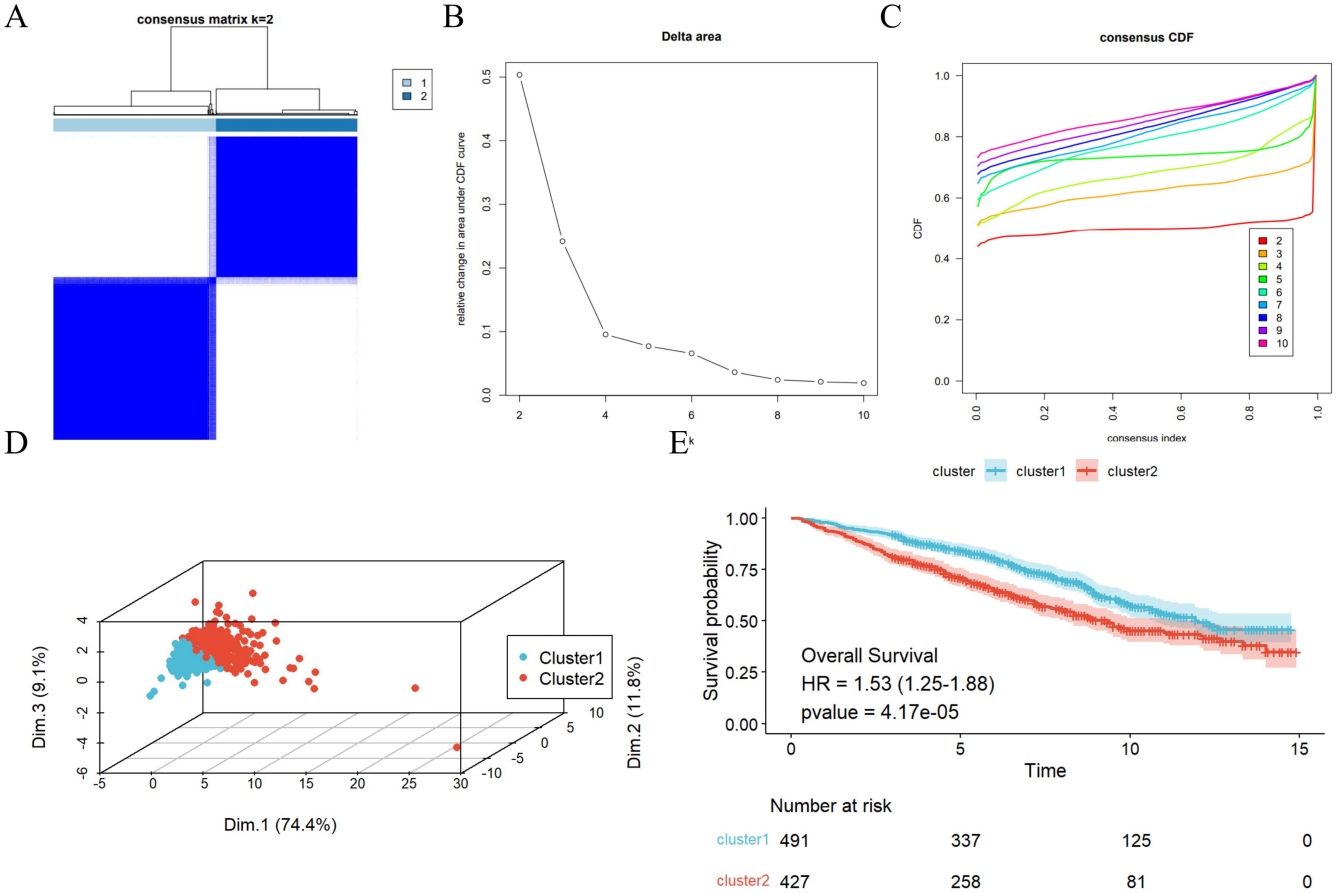
Two inflammation-cluster based NPAR and SIRI. **(A)** Unsupervised cluster analysis of SIRI and NPAR developed two clusters (k = 2); **(B)** Consensus CDF curves when k = 2 to 10; **(C)** Relative alterations in CDF delta area curves; **(D)** Kaplan–Meier plotter of two clusters. ^*^p < 0.05, ^***^p < 0.001; **(E)** 3D Principal Component Analysis delineating the segregation between Cluster C1 and Cluster C2.

The results of the consensus clustering analysis, which utilized the key inflammatory markers NPAR and SIRI, are illustrated in Figures 3A–C. The PAC metric identified the optimal number of clusters as k = 2, leading to the formation of two distinct clusters: C1 (*n* = 491) and C2 (*n* = 427). The principal component analysis (PCA) plot indicates a strong separation between cluster C2 and cluster C1, as depicted in Figure 3E. Furthermore, the Kaplan–Meier (KM) curve analysis assessing all-cause mortality risk for the two inflammatory subtypes revealed that the survival probability for subtype C2 was significantly lower than that for subtype C1 (Figure 3D). An examination of the baseline clinical characteristics for the two subtypes, presented in Table 3, indicated that both age and inflammatory markers were significantly elevated in subtype C2 compared to subtype C1 (*p* < 0.05).

**Table 3:** Characteristics of the study population in two inflammation cluster.

	Cluster			
Characteristic	Overall, *N* = 918^1^	Cluster1, *N* = 491^1^	Cluster2, *N* = 427^1^	*p* value^2^
MORTSTAT				0.004
0	549 (64%)	321 (70%)	228 (57%)	
1	369 (36%)	170 (30%)	199 (43%)	
Age	67.0 (56.0, 77.0)	65.0 (54.0, 75.0)	68.0 (58.0, 78.0)	0.034
Gender				0.3
Female	465 (56%)	247 (54%)	218 (58%)	
Male	453 (44%)	244 (46%)	209 (42%)	
Race				0.042
Mexican American	85 (4.4%)	45 (4.8%)	40 (3.9%)	
Non-Hispanic Black	234 (13%)	147 (16%)	87 (11%)	
Non-Hispanic White	494 (73%)	238 (68%)	256 (78%)	
Other Hispanic	56 (2.8%)	34 (3.0%)	22 (2.6%)	
Other Race	49 (6.9%)	27 (8.7%)	22 (4.8%)	
Education				0.7
Above high school	355 (45%)	190 (47%)	165 (44%)	
Below high school	141 (9.9%)	76 (10%)	65 (9.8%)	
High school	422 (45%)	225 (43%)	197 (46%)	
Income				0.3
Poverty	227 (20%)	125 (19%)	102 (22%)	
Richer	691 (80%)	366 (81%)	325 (78%)	
BMI	29 (25, 34)	29 (25, 33)	29 (25, 35)	0.5
Drinker	608 (69%)	313 (67%)	295 (71%)	0.4
Smoker	259 (28%)	135 (26%)	124 (30%)	0.2
Hypertension	704 (75%)	368 (72%)	336 (78%)	0.2
Diabetes				0.8
Diabetes	343 (33%)	172 (32%)	171 (34%)	
Normal	275 (33%)	153 (34%)	122 (32%)	
Prediabetes	300 (34%)	166 (34%)	134 (34%)	
NLR	2.19 (1.69, 3.06)	1.75 (1.38, 2.06)	3.13 (2.40, 3.93)	<0.001
PLR	123 (94, 155)	106 (84, 131)	140 (114, 188)	<0.001
MLR	0.29 (0.22, 0.40)	0.26 (0.21, 0.33)	0.36 (0.27, 0.47)	<0.001
NPAR	145 (128, 160)	129 (119, 138)	162 (154, 175)	<0.001
SII	525 (361, 730)	390 (286, 510)	711 (564, 957)	<0.001
SIRI	1.26 (0.86, 1.95)	0.96 (0.67, 1.30)	1.82 (1.28, 2.48)	<0.001
AISI	299 (187, 450)	220 (141, 322)	419 (288, 606)	<0.001

^1^*n* (unweighted) (%); Median (Q1, Q3). ^2^Pearson’s X^2^: Rao-Scott adjustment; Design-based Kruskal-Wallis test.

### 3.6 Analysis of the subtype prognostic inflammation risk scoring model

**Figure 4:**
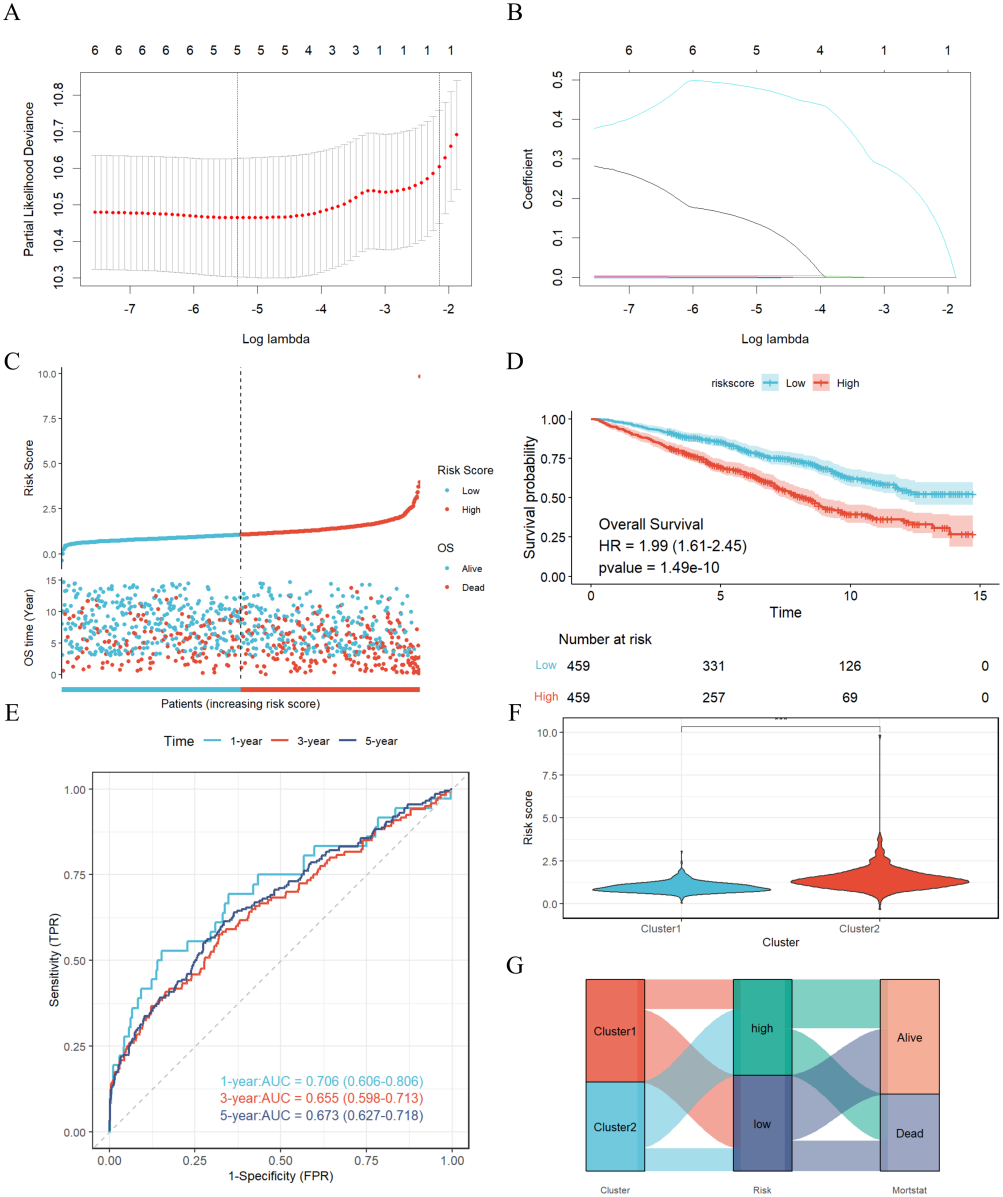
Construction and validation of the risk score model. **(A,B)** Constructed a prognostic model in the through LASSO COX regression analysis. **(C)** Risk scores distribution and survival status of each patient. **(D)** Kaplan–Meier curves for the survival probability of the two subtypes. **(E)** ROC curves illustrated the predictive efficacy of the risk score for 1-, 3-, and 5-year survival; **(F)** The difference in risk scores between two clusters; **(G)** Alluvial diagram of subtype distributions and prognosis of stroke patients.

A risk model was established utilizing LASSO-Cox regression analysis to evaluate the survival risk associated with various subtypes (Figures 4A,B). The risk score is computed using the following formula: Risk score = (0.1543 × NLR) + (0.0017 × PLR) + (0.0041 × NPAR) + (0.4874 × SIRI) − (0.0012 × SII). Subsequently, patients who experienced a stroke were categorized into high-risk and low-risk groups based on the median risk score. Figure 4C illustrates the distribution of risk scores alongside survival duration in stroke patients. The results of the KM analysis presented in Figure 4D indicate that the overall survival time for the high-risk group, characterized by elevated risk scores, is significantly shorter than that of the low-risk group (HR, 1.99 (1.61–2.45), *p* < 0.001), thereby suggesting a poorer prognosis for the high-risk cohort. Furthermore, Figure 4E displays the receiver operating characteristic (ROC) curve, revealing that the area under the curve (AUC) for the time-dependent ROC at 1, 3, and 5 years for the three cohorts is 0.706, 0.655, and 0.673, respectively, which indicates that the prognostic model exhibits commendable predictive performance. Additionally, Figure 4F indicates that Cluster 2 possesses a higher risk score, while Figure 4G presents a Sankey diagram that depicts the distribution of inflammation risk scores, encompassing both the risk scores and survival status across the two subtypes.

### 3.7 Screening of hub prognostic inflammation indicators based on WQS regression analysis and machine learning

**Figure 5:**
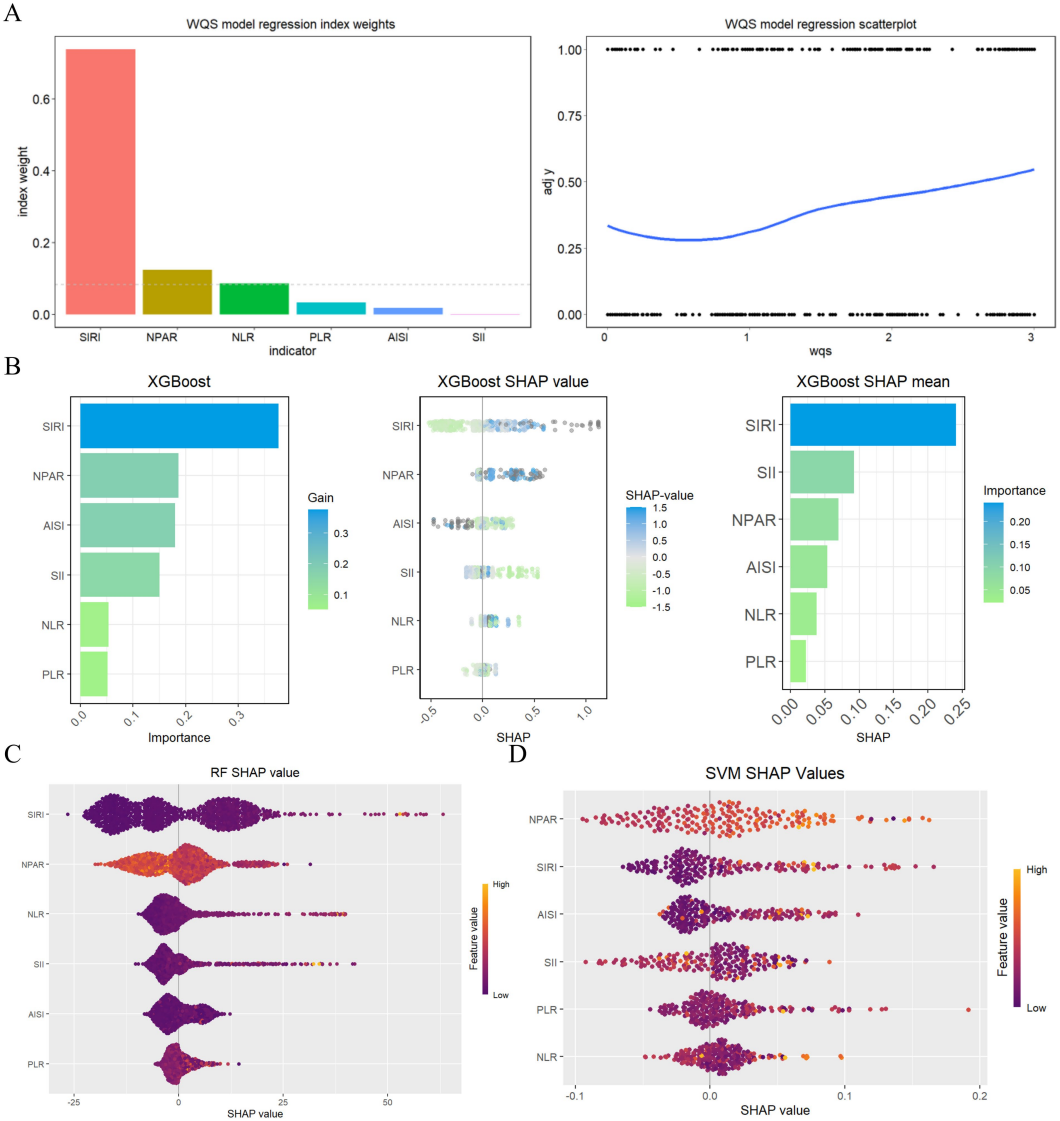
Evaluating the importance of indicators based on WQS analysis, XGBoost, RF, SVMRFE, and COX analysis **(A)** WQS model regression index weights for inflammation indicators on stroke patients; **(B)** Screening of prognostic biomarkers based on XGBoost algorithm; **(C)** Important features selected by random forest algorithm; **(D)** Through SVF-RFE algorithm selects the best indicators.

The weighted quantile sum (WQS) model (Figure 5A) used to evaluate the relationships between six inflammatory markers and the risk of all-cause mortality among stroke patients, revealing a positive association between these inflammatory indicators and mortality risk (slope 0.36, *p* < 0.001). An analysis of the primary contributions of the inflammatory markers within the WQS model indicated that the SIRI had the most substantial effect (73.82%), followed by NPAR (12.42%), NLR (8.61%), and PLR (3.28%). The XGBoost algorithm identified the SIRI, NPAR, AISI, and NLR as the four most significant inflammatory indicators (Figure 5B), while the RF algorithm corroborated these findings, also ranking the SIRI, NPAR, AISI, and NLR as the top four indicators (Figure 5C). Furthermore, the Support Vector Machine Recursive Feature Elimination (SVMRFE) algorithm demonstrated that reducing the number of indicators to five minimized classification errors and maximized accuracy, identifying SIRI, AISI, NLR, NPAR, and SII as key indicators (Figure 5D). In summary, the NPAR and SIRI were ultimately recognized as central prognostic inflammatory indicators.

### 3.8 Construction of a prognostic model for all-cause mortality risk in stroke patients

**Figure 6:**
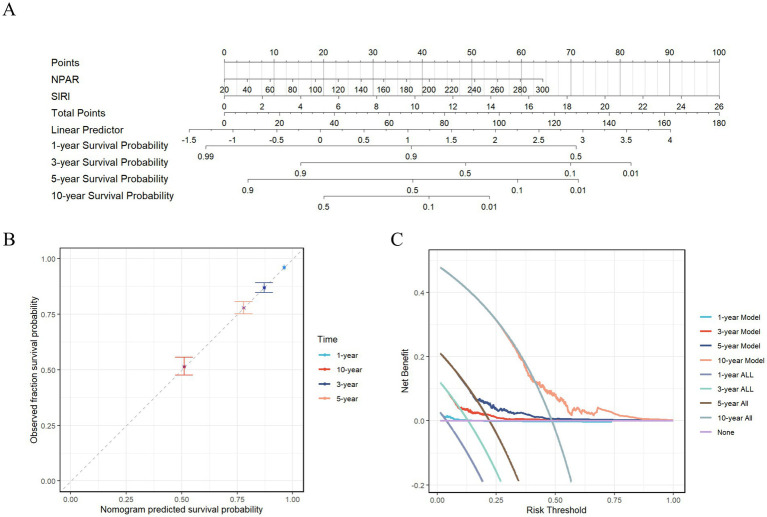
Construction of a prognostic model for all-cause mortality risk in stroke patients. **(A)** The Nomogram containing NPAR and SIRI; **(B)** The calibration plot of nomogram; **(C)** The DCA curves of nomogram.

Based on the evaluation of key prognostic inflammatory markers, NPAR and SIRI were identified for the development of a prognostic nomogram model (Figure 6A). The concordance index (C-index) for this prognostic model was determined to be 0.637 (95%CI, 0.627–0.647). Analysis of the calibration curve demonstrated a strong alignment between the observed and predicted overall survival rates at 1, 3, 5, and 10 years (Figure 6B). Furthermore, decision curve analysis (DCA) revealed that the integrated model, which incorporates these two inflammatory indices, offers the most favorable net benefit for overall survival across the same time intervals (Figure 6C).

## 4 Discussion

The present study investigated the relationship and prognostic significance of six novel inflammatory markers derived from CBC data in relation to all-cause mortality among patients who have experienced a stroke. Our findings revealed a statistically significant positive correlation between the inflammatory markers NPAR and SIRI and the risk of all-cause mortality in this patient population (*p* < 0.001). Consequently, we employed consensus clustering to categorize stroke patients into two distinct inflammatory subtypes. Notably, subtype 2 exhibited markedly elevated levels of inflammatory marker expression and a heightened risk of all-cause mortality when compared to subtype 1 (*p* < 0.001). These results offer valuable insights for the inflammatory risk stratification and management of clinical stroke patients, underscoring the necessity for intensified monitoring and intervention for individuals with elevated levels of SIRI and NPAR, which may ultimately contribute to improved survival outcomes.

This research initially utilized logistic regression and weighted multivariable Logit models to evaluate the associations between six novel inflammatory markers derived from complete blood counts (CBC) and the risk of all-cause mortality among patients who have experienced a stroke. These inflammatory markers, derived from routine CBC tests, reflect the body’s immune activation status and inflammatory burden. Our focus on NPAR, SIRI, and other CBC-based indices was motivated by their cost-effectiveness and universal availability in routine clinical practice and emerging evidence supporting their prognostic value in stroke patients. Our investigation, utilizing data from the NHANES database, assessed the cumulative effects of various novel combined inflammatory indicators, thereby enhancing our understanding of their influence on the prognostic outcomes for stroke patients. The findings indicated that inflammation is a significant factor in the prognosis of stroke patients, particularly highlighting a strong positive correlation between elevated levels of the SIRI and NPAR with an increased risk of all-cause mortality among this population (*p* < 0.001). These results imply that inflammation is a critical component in the pathophysiology of stroke and possesses substantial prognostic value in forecasting all-cause mortality in stroke patients, independent of potential confounding variables such as demographic factors, socioeconomic status, and lifestyle choices. This result aligns with prior studies (3–5). A study (13) involving 1,484 stroke patients indicated that an increased mitochondrial DNA copy number is correlated with a decrease in mortality rates, implying that inflammatory processes may influence the prognosis of individuals who have experienced a stroke. Collectively, these findings support the hypothesis that inflammatory markers can act as prognostic indicators of mortality among stroke patients. Nevertheless, the literature presents conflicting results. For example, a separate investigation involving 1,316 stroke patients found no significant association between the systemic immune-inflammation index and mortality, suggesting that the relationship may differ based on specific patient demographics or the methodologies employed in the studies (14). Additionally, another study suggested that while inflammation is a key factor in stroke, its role in predicting mortality may not be as direct due to the complex interactions of various biological processes involved in stroke recovery (15). These discrepancies may arise from variations in study design, sample size, and the particular inflammatory markers evaluated.

Utilizing the findings from model 3 of the weighted multivariable logistic regression, which accounted for all covariates, we identified the SIRI and NPAR as significant inflammatory indicators. We then employed consensus clustering to categorize stroke patients into distinct inflammatory subtypes. The results of the clustering analysis revealed two separate inflammatory subgroups, with subgroup 2 demonstrating markedly elevated levels of inflammatory markers and an increased risk of all-cause mortality in comparison to subgroup 1 (*p* < 0.001). Following this, we developed an inflammatory risk scoring model for stroke patients through LASSO regression analysis to evaluate survival risks across the identified subtypes. The findings indicated that the risk score for subgroup 2 was significantly greater than that for subgroup 1, with subgroup 2, classified as high-risk, exhibiting a notably reduced overall survival compared to the low-risk group (HR, 1.99 (1.61–2.45), *p* < 0.001). This suggests that the high-expression subtype 2 is associated with a poorer prognosis. These results provide a valuable reference for the inflammatory risk stratification management of clinical stroke patients, highlighting the necessity for enhanced monitoring and management of individuals with elevated levels of SIRI and NPAR, which may contribute to improved patient survival outcomes.

Finally, we employed a variety of statistical methods—including weighted quantile sum (WQS) regression and three machine learning techniques—to identify key inflammatory markers that influence stroke prognosis. The WQS model demonstrated a significant association between six inflammatory markers and the risk of all-cause mortality in stroke patients, revealing a positive correlation between these inflammatory indicators and mortality risk (slope = 0.36, *p* < 0.001). Integrating these various statistical results, NPAR and SIRI were ultimately identified as hub prognostic inflammatory indicators. A prognostic nomogram model was subsequently constructed based on these indicators. Evaluation of the model’s performance through calibration curves and decision curve analysis (DCA) revealed that the comprehensive model incorporating both NPAR and SIRI offered the best net benefit for predicting overall survival in stroke patients at 1, 3, 5, and 10 years. These statistical methods, from different analytical perspectives, enhanced the robustness of our conclusions and emphasized the importance of inflammatory markers as potential intervention targets in managing the mortality risk of stroke patients, providing a new perspective on the prognostic value of inflammatory markers in stroke patients. It reminds us to pay special attention to NPAR and SIRI, as both serve as reliable prognostic indicators, with their elevation often accompanying a high risk of mortality in stroke patients, consistent with previous studies. For example, Cui et al.’s (16) retrospective study found that NPAR was positively correlated with adverse functional outcomes (OR, 2.76 (1.52–5.03), *p* = 0.001); Yang et al.’s (17) study shown that NPAR was independently associated with the risk of recurrence within 3 months (OR 9.71 (3.05–31.62), *p* < 0.001); Chen et al.’s study (18) indicated that higher NPAR still had significant predictive ability for 30-day all-cause mortality (HR, 1.45 (1.05–2.00), *p* < 0.05); Huang et al.’s (19) research displayed that SIRI exhibited strong predictive ability in identifying adverse outcomes and stroke-associated pneumonia, with higher SIRI values typically associated with negative endpoints. However, two critical gaps are addressed in our work. Our study utilized the NHANES database to analyze not only the long-term prognostic outcomes of stroke patients but also to stratify patients based on inflammatory risk, providing a reference for healthcare professionals in identifying different inflammatory subtypes in stroke patients and offering personalized treatment.

Our inflammatory subtype prognostic model is positioned as a complementary decision-support tool to established stroke prognostic scores (e.g., ASTRAL, iScore), specifically designed to provide inflammation-enhanced risk stratification. We propose a stepped integration protocol in clinical practice—initial baseline risk assessment using conventional scores (e.g., THRIVE) followed by individualized adjustments based on inflammatory subtypes, including anti-inflammatory therapy intensification and monitoring frequency stratification (6-month intervals for low-risk vs. monthly for high-risk subgroups). Nonetheless, it is important to recognize the limitations of this study. Firstly, the dependence on the NHANES database may introduce biases typical of observational studies, as there may be unmeasured confounding variables despite our comprehensive statistical adjustments. Secondly, single-timepoint biomarkers cannot capture post-stroke inflammatory dynamics. Third, the lack of stroke subtyping data prevented us from exploring potential differences in inflammatory profiles between ischemic and hemorrhagic stroke, which warrants further investigation in cohorts with detailed phenotyping. To address these limitations, we have initiated a prospective validation study (Haiyan Stroke Cohort) featuring with dynamic multi-timepoint monitoring. This protocol has been approved by Haiyan People’s Hospital Ethics Committee (2024-52) and the preliminary results will be reported in 2026.

## 5 Conclusion

In conclusion, our study identified a significant positive correlation between the inflammatory markers NPAR and SIRI and all-cause mortality among stroke patients. Consequently, we developed various inflammatory subtypes and prognostic models based on these findings. This research not only underscores the relevance of these biomarkers as potential prognostic indicators but also establishes a comprehensive framework for clinical risk stratification and patient management. By integrating inflammation assessment into standard clinical practice, we can enhance patient outcomes and facilitate the development of targeted interventions, which are essential for addressing the morbidity and mortality challenges associated with stroke. Specifically, for patient populations at elevated risk of inflammation, personalized treatment strategies can markedly improve prognosis and mitigate the long-term burden related to stroke.

## Data Availability

The original contributions presented in the study are included in the article/supplementary material, further inquiries can be directed to the corresponding authors.
